# Evaluation of the Antioxidant and Anticancer Activities of Hydroalcoholic Extracts of *Thymus daenensis* Čelak and *Stachys pilifera* Benth

**DOI:** 10.1155/2022/1924265

**Published:** 2022-03-30

**Authors:** Zahra Barmoudeh, Maryam Tajali Ardakani, Amir Hossein Doustimotlagh, Hassan Bardania

**Affiliations:** ^1^Student Research Committee, Yasuj University of Medical Sciences, Yasuj, Iran; ^2^Cellular and Molecular Research Center, Yasuj University of Medical Sciences, Yasuj, Iran; ^3^Medicinal Plants Research Center, Yasuj University of Medical Sciences, Yasuj, Iran; ^4^Department of Clinical Biochemistry, Faculty of Medicine, Yasuj University of Medical Sciences, Yasuj, Iran; ^5^Clinical Research Development Unit, Imamsajad Hospital, Yasuj University of Medical Sciences, Yasuj, Iran

## Abstract

Herein, the effects of hydroalcoholic extracts of *Thymus daenensis* Celak (TDC) and *Stachys pilifera* Benth (SPB) plants on HepG2 cell line were investigated by using different analyses. Cytotoxicity and apoptosis of extracts were investigated by MTT method, AnnV/PI apoptosis assay, and their antioxidant capacity was evaluated by total thiol and glutathione peroxidase (GPX) assay. The results revealed that the SBP extract was more cytotoxic compared with the TDC extract and increased over time (128.49 *µ*g/mL vs 107.11 *µ*g/mL IC_50_ values for 24 and 72 h, respectively). Although, AnnV/PI apoptosis assay showed apoptosis induction for both extracts, but the caspase-3 activity assay revealed that TDC extract significantly increased caspase-3 activity compared with the control and SPB extract. Increasing the activity of GPX by SPB extract revealed that it has high antioxidant capacity. In conclusion, the TDC and SPB with high antioxidant capacity have high cytotoxicity against HepG2 cancer cells and have high capability as a medicinal plant.

## 1. Introduction

Hepatocellular carcinoma (HCC) is the fourth leading cause of all deaths as a result of cancer, in the world [1]. This type of cancer, which usually presents in an advanced stage, has a poor prognosis [2]. The prevalence of this type of cancer varies around the world but is very high in developing countries. HCC is the fourth and ninth most common cancers in men and women, respectively [3]. HCC usually occurs in the background of cirrhosis for a variety of reasons, including alcoholism, hepatitis B virus, hepatitis C virus, hemochromatosis, Wilson's disease, type 2 diabetes, and hemophilia [4]. Hepatitis C and hepatitis B virus are still the most important risk factors for HCC [4]. Studies show that oxidative stress is involved in liver cancer [5], but its mechanism, and above all, its effects are unclear.

Reactive oxygen species (ROS) including hydrogen peroxide (H_2_O_2_), superoxide anion (O_2_^−^), and hydroxyl radical (HO^.^), which are mainly produced through breathing, metabolism, or inflammation, can motivate mutations in larger genomic sites or lesions. Also, H_2_O_2_ is considered as a signaling molecule which can balance inflammation, multiplication, segregation, protection, autophagy, metastasis, and metabolic pathways. The action of these pathways can determine the malignancy [6]. Within a healthy cell, there is a proper balance between peroxidants and antioxidants. Loss of balance between peroxidants and antioxidants causes oncogenesis and progression of the HCC tumor [7].

There are many efficient chemical drugs for treatment of HCC tumor, but they have many obstacles including mutations, carcinogens, gastrointestinal disorders, and kidney failure. Therefore, many studies are performing to find new drugs with fewer side effects. Medicinal plants have attracted a lot of attention due to their constituents [8, 9]. They can effect on different stages of the onset and growth of cancerous cells [10]. Here, we studied the effect of hydroalcoholic extracts of *Thymus daenensis* Celak (TDC) and *Stachys pilifera* Benth (SPB) on human liver cancer cell line (HepG2).

TDC from mint family (Lamiacae) grows wildly in central and northwestern of Iran [11]. The leaves and flowering parts of TDC are traditionally used for diverse medical purposes, for example as an antispasmodic, anticonvulsant, cough, bloating, anti-inflammatory, analgesic, antifungal, and nasal decongestant [12]. This plant, with high antioxidant properties, contains tannins, flavonoids, and glycosides. Its essential oil includes thymol (73.9%), carvacrol (6.7%), p-cymene (4.6%), *γ*-terpinene (1.4%), and borneol (1.1%) [13, 14].

SPB (from mint family, Lamiaceae) is mostly distributed in temperate and tropical regions [15]. This plant contains phenylethanoid glycosides, saponins, flavonoids, terpenoids, diterpenes, and steroids [16]. The aerial parts of this plant in the form of herbal tea are used orally to treat infectious, respiratory, rheumatoid, and inflammatory disorders [17]. As a traditional medicine, plants of this genus have been used to treat genital tumors, sclerosis of the spleen, inflammatory tumors, cough, antianoxic surgery, wound healing, treatment of abdominal pain, and as antiseptic, antispasmodic, and antipyretic [18]. Also, antibacterial, anti-inflammatory, antitoxic, antinephritic, antioxidant, antihepatitis, hepatoprotective, and anticontractile effects of *Stachys* species have been proven in different studies [19–21].

Herein, we evaluated the effect of hydroalcoholic extracts of TDC and SPB on HepG2 cell line by using different methods including MTT and AnnV/PI apoptosis assays.

## 2. Materials and Methods

### 2.1. Materials

Fetal bovine serum (FBS), Roswell Park Memorial Institute (RPMI) 1640 medium, penicillin, streptomycin, and trypsin were provided by Gibco BRL (Grand Island, NY, USA). Dimethyl sulfoxide (DMSO), 3-(4,5-dimethylthiazol-2-yl)-2,5-diphenyltetrazolium bromide (MTT), Pen-Strep (penicillin-streptomycin), ethanol, 5,5-dithiobis-2-nitrobenzoic (DTNB), ethylenediaminetetraacetic acid (EDTA), and cisplatin were purchased from Sigma Chemical Co. (St. Louis, MO, USA). Other materials such as trichloroacetic acid (TCA), 2,4-dinitrophenylhydrazine (DNPH), guanidine hydrochloride and 2,4,6-tris(2-pyridyl)-s-triazine (TPTZ) were purchased from Merck (Germany). All chemicals and reagents were used without any further purification. All procedures were accepted by the Ethics Committee of Yasuj University of Medical Sciences (ethical code: IR.YUMS.REC.1398.114).

### 2.2. Plant Materials

TDC and SPB plants were collected from the mountains around Yasuj, the capital of Kohgiluyeh, and Boyer-Ahmad provinces in the spring. The TDC and SPB plants were authenticated by Dr. A. Jafari from Department of Botany, Center for Research in Natural Resource and Animal Husbandry, Yasuj University, Yasuj, Iran, where a voucher specimen (herbarium no. 496 and herbarium no. 1897) were deposited, respectively. The plants were dried and stored under appropriate conditions, away from sunlight, and then powdered. The extraction of 50 g dried powder was prepared by 70% methanol using maceration method for 72 hours. Then, the mixture was filtered using a Whatman filter paper and dried in an incubator at 37°C. In our previous study, the chemical composition of SPB and TDC extracts were determined by GC-MS analysis [21]. Also, Alizadeh et al. determined that the main components of the TDC oil were thymol (66.62–71.49%), p-cymene (5.52–7.12%), and *β*-caryophyllene (3.91–4.09%) [11].

### 2.3. *In Vitro* Cytotoxicity Assay

Hepatocellular carcinoma (HepG2) cell line was provided by Pasteur Institute of Iran (Tehran, I.R. Iran) and cultured in RPMI 1640 medium containing 10% (v/v) FBS, 100 unit/mL penicillin, and 100 *μ*g/mL streptomycin. The cells were incubated at 37°C in a humidified atmosphere (90%) with 5% CO_2_. MTT (4,5-dimethyl diphenyl tetrazolium bromide) assay was used to examine the effect of TDC and SPB plants extract on HepG2 cell viability [22, 23]. The cells were seeded in a 96-well plate with the density of 8 × 10^3^ cells per well and incubated for 24 hours. Then, they were treated with different concentrations (50, 100, 250, 500, 700, and 1000 *μ*g/mL) of plants extract and incubated for 24 hours in an incubator. After that, the culture medium was replaced with 100 *µ*l of MTT solution (0.5 mg/mL). After 4 hours' incubation at 37°C, MTT solution was removed and DMSO solution was added to the wells to dissolve formed formazan crystals for 15 min. Finally, the absorption of the samples at 570 nm was monitored by using an ELISA reader (BioTek ELx800; BioTek Instruments Inc., Winooski, VT, USA). The cell viability (%) was computed:

(1) $$\begin{array}{c} \left(\frac{\text{Control OD}}{\text{OD sample}}\right)\times 100=\text{cell viability}. \end{array}$$

### 2.4. Annexin-V/Propidium Iodide (PI) Apoptosis Assay

The apoptotic effect of hydroalcoholic extracts on cells were studied by an annexin-V FITC/propidium iodide (PI) double-staining method (eBioscienceThermo Fisher Sci.; USA). Briefly, HepG2 cells were seeded in 6-well culture plates (2.5 × 10^5^ cells/well) in RPMI containing 10% FBS and incubated at 37°C for 24 h. According to MTT data, the cells were then treated by plant extracts (75 *μ*g/mLand 100 *μ*g/mL of SPB and TDC extracts, respectively) and incubated for 24 hours. After that, the cells were detached and transferred to a falcon tubes and the supernatant was discarded after centrifugation. Next, 500 *μ*l of binder buffer, 5 *μ*l of Annexin-V, and 5 *μ*l of PI were added to the treated cells by and the cells were incubated for 20 minutes in the dark at 25°C. Finally, the vials containing stained cells were examined by using a Becton Dickinson FACS Calibur flow cytometer.

### 2.5. Caspase-3 Activity Assay

100 and 200 *µ*g/mL of TDC and 75 and 150 *µ*g/mL of SPB extracts were added to HepG2 cells with the density of 8 × 10^4^ cells/mL and incubated for 24 h. Then, the cells were collected and the caspase-3 activity was used for determining the apoptotic effect of plants extract by the aid of a caspase-3 activity fluorometric assay kit (Abcam, Cambridge, UK). The caspase-3 activity assay was performed based on the manufacturer's instructions.

### 2.6. Oxidative Stress Markers

According to MTT results and like apoptosis assays, at a primary experiment, we used two concentrations of plant extracts for examining oxidative stress markers. Accordingly, 200 *µ*g/mL of TDC, and 150 *µ*g/mL of SPB were selected for experiments related to oxidative stress markers. Therefore, HepG2 cells were seeded in 6-well culture plates (2.5 × 10^5^ cells/well) and incubated for 24 h. Then, the cells were treated with 200 *µ*g/mL of TDC, and 150 *µ*g/mL of SPB and incubated for 24 h. Then, we used lysed cells to measure the ferric reducing antioxidant power (FRAP) and total thiol as well as GPX activity.

### 2.7. Ferric Reducing Antioxidant Power (FRAP) Assay

The FRAP assay was performed based on the previous study [24]. In this method, Fe-TPTZ complex is formed when the ferric ions (Fe3+) has been reduced and converted to ferro-ions (Fe2+) in acidic pH. In the presence of TPTZ (tripyridyl-s-triazine), the reaction color was changed to blue. The color intensity was monitored by an ELISA reader (BioTek ELx800; BioTek Instruments Inc., Winooski, VT, USA) at 593 nm. This reaction is nonspecific, and any molecule capable of regenerating the ferric ion under the above conditions can participate. So, all the measurements were performed against the reagent blank.

### 2.8. Total Thiols (T-SH)

The ability of thiols to oxidize 5,5-dithiobis-2-nitrobenzoic (DTNB) was evaluated by measuring the absorbance at 412 nm [25]. 25 *μ*l of the sample (lysed treated HepG2 cells) was added to Tris buffer (150 *μ*l). 790 *μ*l of absolute methanol and 20 *μ*l of DTNB (10 mM in methanol) were then added to the above mixture followed by incubation for 20 min at 25°C. Afterward, the tubes were centrifuged at 3000 × g for 10 min and its supernatant separated. After that, each sample absorbance was measured against DTNB blank and blank tube at 412 nm. The total T-SH was considered through the molar absorption ratio of 13600 (M^−1 ^cm^−1^).

### 2.9. Glutathione Peroxidase

The effects of plant extracts on enzymatic antioxidant defenses were studied by GPX activity. The GPX activity was measured using a commercial chemical colorimeter color measurement kit (ZellBio GmbH, Ulm, Germany) using spectrophotometry.

### 2.10. Statistical Analyses

All experiments were performed in triplicate and the results were expressed as the mean ± SEM. Analysis of variance (ANOVA) was used to clarify the significance differences between groups followed by Tukey post hoc analysis. *P* value≤0.05 was assumed as statistically significant.

## 3. Results

**Figure 1:**
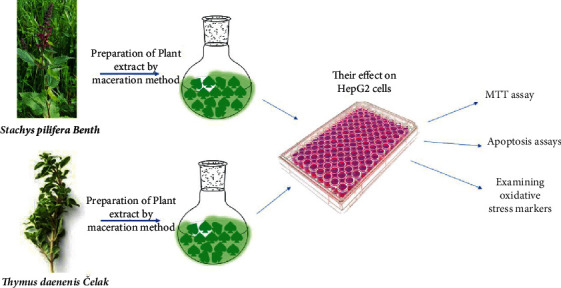
The experimental design of the study.

Figure 1 shows the experimental design of the study.

### 3.1. *In Vitro* Cytotoxicity Assay

**Figure 2:**
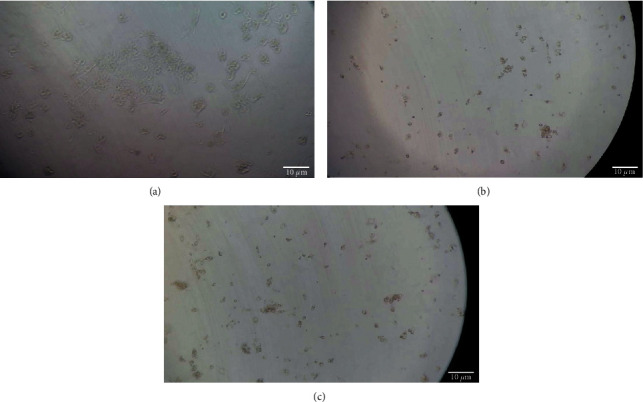
Optical microscope images of HepG2 cell lines. (a) Untreated. (b) Treated by 500 *µ*g/ml TDC. (c) Treated by 500 *µ*g/ml SBP plants extracts. Firstly, 8 × 10^3^ cells per well were seeded in a 96-well plate and incubated for 24 hours, after that treated by plant extracts. The assay was repeated three times. Scale bar = 10 *µ*m.

*In vitro* cytotoxicity of plants extract was studied through MTT assay. The results revealed that the SBP extract was more cytotoxic compared with the TDC extract. In addition, the results revealed that cytotoxicity effect of the SBP extract increase over time (128.49 *µ*g/mL vs 107.11 *µ*g/mL IC_50_ values for 24 and 72 h, respectively) (Table 1) (Figure 2). Figure 2 shows the morphological properties of untreated cells and treated cells. The cells have deformed by treating with extracts (Figure 1).

### 3.2. Apoptosis Assay

**Figure 3:**
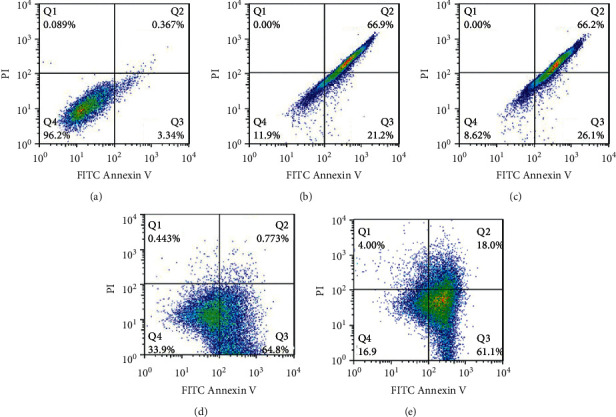
The apoptosis assay in HepG2 cells after treatment for 24 h of different concentrations of (a) free media, (b) 100 *µ*g/mL and (c) 200 *µ*g/mL *T. daenensis* Celak extract, and (d) 75 *µ*g/mL and (e) 150 *µ*g/mL *S. pilifera* Benth extract. Q1 represents the necrosis cells, Q2 the late apoptosis cells, Q3 the cells in early apoptosis, and Q4 the viable cells.

Flow cytometry was the technique used for detection of Annexin-V/propidium iodide (PI) apoptosis assay to study the apoptotic effect of plant extracts on HepG2 cell line. The results showed that the TDC extract had more late apoptotic effect on cells for both 100 and 200 *µ*g/mL concentrations (66.9% vs 65.2%, respectively) (Figures 3(a)–3(c)). Furthermore, increasing the SBP extracts concentration from 75 to 150 *µ*g/mL induced apoptosis. Early apoptosis effect was 64.8% vs 61.1% for 75 and 150 *µ*g/mL concentration of SBP and late apoptosis effect was 0.773% vs 18.0% for 75 and 150 *µ*g/mL concentration (Figures 3(d) and 3(e)).

### 3.3. Caspase-3 Activity Assay

**Figure 4:**
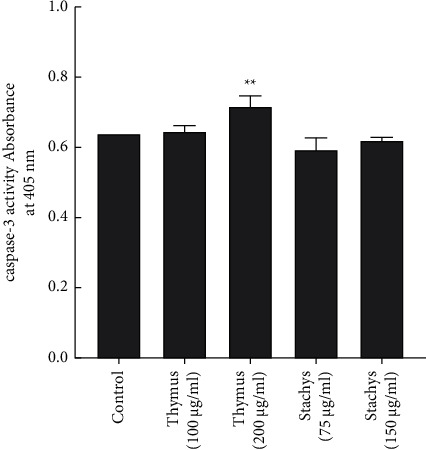
Effect of the *S. pilifera* Benth and *T. daenensis* Celak extracts for 24 h on the caspase-3 activity in HepG2 cells (^*∗∗*^*P* < 0.01, in comparison to the control).

Caspase-3 enzyme has a crucial role in the terminal process of apoptosis [26, 27]. As illustrated in Figure 4, 200 *µ*g/mL TDC extract greatly induced the increase of caspase-3 activity compared with the control group (*P* < 0.01). Other groups such as TDC extracts with lower concentration (100 *µ*g/mL) and both concentrations (75 and 150 *µ*g/mL) of SBP did not significantly change in caspase-3 activity compared with untreated cells.

### 3.4. FRAP Assay

**Figure 5:**
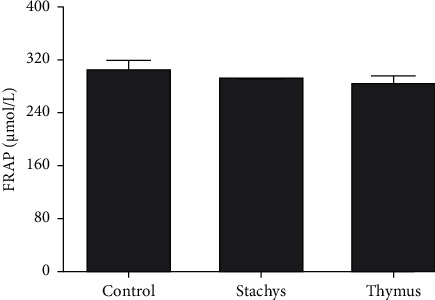
The total antioxidant activity of *S. pilifera* Benth and *T. daenensis* Celak extracts for 24 h in HepG2 cells.

The FRAP values of adequate concentrations of 200 *µ*g/mL of TDC and 150 *µ*g/mL of SBP revealed that the reducing power of extracts was not significantly changed compared to free media (Figure 5).

### 3.5. Estimation of Total Thiols

**Figure 6:**
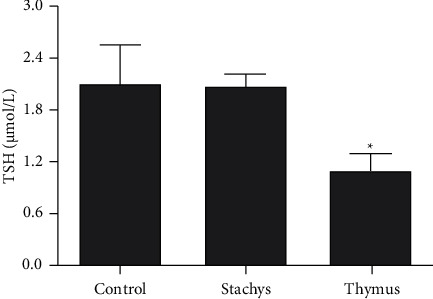
Effect of *S. pilifera* Benth and *T. daenensis* Celak extracts on total thiols (T-SH), for 24 h in HepG2 cells (^*∗*^*P* < 0.05, in comparison to the control).

Although the TDC (200 *µ*g/mL) extract induced a significant reduction in the level of T-SH (*P* < 0.05), the SBP (150 *µ*g/mL) extract had not significant effect on the T-SH level (Figure 6).

### 3.6. Glutathione Peroxidase

**Figure 7:**
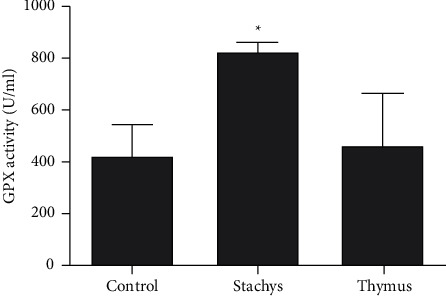
*S. pilifera* Benth and *T. daenensis* Celak extracts on glutathione peroxidase (GPX) activity for 24 h in HepG2 cells (^*∗*^*P* < 0.05, in comparison to the control).

The antioxidant enzyme activity of two TDC (200 *µ*g/mL) and SBP (150 *µ*g/mL) extracts was assessed by evaluating the activity of glutathione peroxidase (GPX). The SBP extract significantly enhanced the GPX activity (*P* < 0.05) compared with the control group, while the TDC extract had no significant effect (Figure 7).

## 4. Discussion

HCC is the most popular primary malignancy of the liver and the third deadliest human cancer in the world [28]. Common treatments for cancer include chemotherapy, radiotherapy, and surgery. They are very costly economically and cause diverse damage to major organs including heart, lung, and kidney. They also could weaken the immune systems which lead to reduction of the life quality of and discourage patients from following drug protocols that ends to the cancer progression [29].

An appropriate anticancer drug should be able to kill cancerous cells without side effects on the normal cells. These ideal conditions are achieved by inducing apoptosis on cancer cells. Many common medicines are derived from plants. Understanding the mechanisms involved in the development of cancer is important for advancing therapies for the treatment of neoplasms. A series of mutations in cells make them resistant to death stimuli and apoptosis. Therefore, the use of apoptosis-inducing constituents of medicinal plants is one of the main aims of cancer treatment. These components can kill cancer cells via the induction of apoptosis through different mechanism [30, 31].

In the recent years, the use of plant compounds to prevent and intervene in various stages of carcinogenesis has attracted more attention. Plant polyphenols are one of the most effective cancer-inhibiting compounds by interfering with multiple intracellular signals, angiogenesis, metastasis, and less systemic effects [28]. The combination of antioxidants having anti-inflammatory properties can be an anticancer agent because of the fact that tumor progression is closely linked to inflammation and oxidative stress [32]. Last reports showed that SBP consists of a rich source of flavonoids. Also, the methanolic extract of this plant have antioxidant and antitumor properties due to flavonoids and phenolic compounds [33, 34]. The goal of the present study was to evaluate the anticancer activity of SBP and TDC hydroalcoholic extracts on HepG2 cancer cell line. Coa et al. found that flavonoids with high antioxidant properties have cytotoxic effects on cancerous cells. In addition, it was showed that pure flavonoids also have anticancer activity against different human cancers, such as hepatoma (HepG2 cells) and cervical cancer (Hela cells) as well as breast cancer (MCF-7 cells) [35]. Jassbi et al. studied the antioxidant, cytotoxic, and antimicrobial effects of different species of *Stachys* on cancer cell lines and showed that *Stachys* plant extracts reduced the proliferation of breast and blood cancer cells [31]. Panahi et al. demonstrated the cytotoxic and antiproliferative effects of SBP on the colorectal cell line by apoptosis induction [36]. Based on the final results of this study, SBP methanolic extract had significant cytotoxic effects on HepG2 compared to control.

On the other hand, TDC has many applications in traditional medicine due to its flavonoids and phenolic compositions such as thymol and carvacrol [37]. The results of the MTT assay revealed the cytotoxic effect of hydroalcoholic extracts of TDC on HepG2 cancer cells. These results are in alignment with the research of Sadeghi et al. and Samani et al. showing that the TDC extract inhibits the growth of MCF-7 cell line *in vitro* [38, 39].

Flow cytometric results showed that SBP and TDC extracts may have apoptotic properties. These results are consistent with results of Singh et al. showing that plant extracts and constituents can induce apoptosis in cancer cells [40]. Greenwell et al. identified cytotoxic polyphenols as apoptotic inducers and anticancer properties for a wide range of cancer cells [35]. Tavakkoli et al. showed that *Carthamus*, *Salvia*, and *Stachys* plant extracts preserve nerve cells against apoptosis caused by oxidative stress [41].

*Stachys* may be considered as good sources of natural antioxidants for medicinal uses [42]. Different ROS compounds such as hydroxyl radicals, peroxides, and superoxides can be produced within normal cell during normal metabolic processes. Overproduction of ROS or insufficiency in the normal cell antioxidant defense system (or both) causes the cell to go through the oxidative stress [43]. Overproduction of ROS can be associated with the onset and development of many cancers in cellular processes. Oxygen-activated species are involved in several cancer cell signaling cascades such as cell growth, angiogenesis, metastasis, glucose metabolism, differentiation, and inflammation. As an oncogenic agent, they play a critical role in the onset, growth, progression, invasion, and metastasis of cancer [44]. Bardi et al. revealed that *Zingiber officinale* extract can have an anticancer effect through replacing the function of SOD, GPX, and catalase in scavenging ROS causing oxidative damage to cells [45].

## 5. Conclusion

The cytotoxicity and apoptotic effect of SBP and TDC aerial part extracts were assessed on HepG2 cell line for the first time in this study. SBP and TDC extracts can be considered as agents with inhibitory effect against hepatocellular carcinoma cells. Therefore, this study provides a beneficial scientific basis for identifying the active ingredients of these extracts and the mechanisms involved in their lethal effects as potential anticancer compounds.

## Figures and Tables

**Table 1:** Calculated IC_50_ values for both TDC and SBP plant extracts.

	IC_50_ (*µ*g/mL) for 24 h	IC_50_ (*µ*g/mL) for 48 h	IC_50_ (*µ*g/mL) for 72 h
*T. daenensis* Celak	203.6 ± 14	210.2 ± 12	223.7 ± 16
*S. pilifera* Benth	128.5 ± 7	109.7 ± 5	107.1 ± 4

## Data Availability

The data supporting the findings of this study are available within the article.
